# Improving disclosure of medical error through educational program as a first step toward patient safety

**DOI:** 10.1186/s12909-017-0880-9

**Published:** 2017-03-04

**Authors:** Chan Woong Kim, Sun Jung Myung, Eun Kyung Eo, Yerim Chang

**Affiliations:** 1 Department of Emergency Medicine, Choong Ang University College of Medicine, Seoul, Republic of Korea; 2 Seoul National University College of Medicine, Office of Medical Education, 103 Daehak-ro, Jongno-gu, Seoul, 110-799 Republic of Korea; 3 Soonchunhyang University Bucheon Hospital, Bucheon, Republic of Korea; 4 Department of Surgery, Dankook University Hospital, Cheonan, Chungcheongnam-do Republic of Korea

**Keywords:** Medical error disclosure, Communication, Simulation, Medical education

## Abstract

**Background:**

Although physicians believe that medical errors should be disclosed to patients and their families, they often hesitate to do so. In this study, we assessed the effectiveness of an education program for medical error disclosure.

**Methods:**

In 2015, six medical interns and 79 fourth-year medical students participated in this study. The education program included practice of error disclosure using a standardized patient scenario, feedback, and short didactic sessions. Participant performance was evaluated with a previously developed rating scale that measures error disclosure performance on five specific component skills. Following education program, we surveyed participant perceptions of medical error disclosure with varying severity of error outcome and their satisfaction with the education program using a 5-point Likert scale. We also surveyed the change of attitude or confidence of participants after education program.

**Results:**

The performance score was not significantly different between medical interns and medical students (*p* = 0.840). Following the education program, 65% of participants said that they had become more confident in coping with medical errors, and most participants (79.7%) were satisfied with the education program. They also indicated that they felt a greater duty to disclose medical errors and deliver an apology when the medical error outcome is more severe.

**Conclusions:**

An education program for disclosing medical errors was helpful in improving confidence in medical error disclosure. Extending the program to more diverse scenarios and a more diverse group of physicians is needed.

**Electronic supplementary material:**

The online version of this article (doi:10.1186/s12909-017-0880-9) contains supplementary material, which is available to authorized users.

## Background

Disclosure of medical errors to patients and families is an important part of patient-centred medical care and essential requirement for maintaining trust. Many countries have established error disclosure as standards of practice or legislation [1, 2]. In the United States, the National Quality Forum issued an evidence-based safe practice guideline on the disclosure of serious unanticipated outcomes [3], which recommended providing an explanation to patients and their families about what happened, describing the potential implications caused by the error, giving a firm promise to examine what would occur and feedback about findings of the examination, and offering a physician’s apology or expression of regret. [3]

Although most physicians think that they should disclose medical errors to patients, they feel uncomfortable to do so due to fear of litigation and declaring medical error to a patient [4–6]. Research demonstrates that disclosure of error is uncommon, with roughly 30% being disclosed [7–12].

Educating physicians and other health care providers is the first step to creating a culture of transparency [13]. This study presents an educational program on the disclosure of medical error and it’s effectiveness on the attitudes regarding the disclosure of medical errors.

## Methods

### Study design

The education program was designed to improve student understanding and performance of medical error disclosure. It includes practice in error disclosure using a standardized patient (SP), facilitated reflection, feedback, and short didactics for wrap-up.

### Scenario

The error scenario used for simulation was medication error in an outpatient clinic, involving unintended medication to a patient who had a medical history of drug allergy. An additional file shows scenario in more detail (see Additional file 1). The case scenario was developed by an emergency medicine doctor and reviewed for validity by two physicians (one internal medicine doctor and one emergency medicine doctor).

### Participants and simulation program

Seventy-nine fourth-year medical students at Seoul National University College of Medicine (SNUCM; Seoul, South Korea), three medical interns at Seoul National University Hospital (SNUH), and three medical interns at Chung-Ang University Hospital (CUH) participated. The 79 fourth-year medical students were divided into two groups, and five students in each group had a chance at a direct SP encounter. Students rotated through the physician role and observer role. Students who did not get the chance to play the physician role also participated in the end-of-case debriefings with the SP and faculty. For the six medical interns, every intern had the chance to have a direct SP encounter one by one. After the SP encounter, all medical interns discussed the case and gave feedback.

### Measures

To evaluate the participants’ abilities (who directly encounter the SP) to disclose medical error, the faculty used a rating scale based on components previously developed by Chan et al. [14] The scale is based on studies that have examined what patients would want to know about medical errors in their care [15], and it has previously been used in a study of error disclosure by surgeons and internal medicine residents [14, 16]. The components comprise the following (see Additional file 2): (1) explanation of the medical facts regarding the error, (2) honesty and truthfulness, (3) empathy, (4) explanation of steps taken to prevent future errors, and (5) general communication skills. The faculty rated participant performances in five categories using a 5-point Likert scale, and an average score of five categories was taken as the total score.

### Questionnaire

Following the SP encounter and debriefing session, we asked participants to answer a questionnaire dealing with three clinical vignettes describing medical error. The vignettes were developed by one internal medicine doctor and two emergency medicine doctors using existing medical error case and experience and reviewed for validity by two physicians (one internal medicine doctor and one surgery doctor). The vignettes differed in severity of outcome from the medical error and involved intrathecal vincristine injection error (case 1), doctor’s prescription error (case 2), and a fall without any visible trauma (case 3). An additional file shows scenario in more detail (see Additional file 3). These surveys were modelled after an existing survey on ethical attitudes and practices and tested [17, 18]. The participants were asked to imagine that they had made the errors, to respond to questions about their likely disclosure practices and attitudes: 1) How would you manage this situation? (essay); 2) Would you apologize to the patient and family? (scored on a Likert scale with 6 anchors: 1 = strongly disagree; 2 = disagree; 3 = disagree a little; 4 = agree a little; 5 = agree; and 6 = strongly agree); and 3) Please provide a way to prevent this situation (essay). We also asked participants to register their satisfaction with the education program using a 5-point Likert scale and the change of attitude or confidence after education program.

### Statistical analysis

We performed descriptive statistics and two-sample proportion comparison test. We used Pearson’s chi-squared test to measure association and used Fisher’s exact test when expected values were <5. The analysis of variance (ANOVA) was used to compare means. Statistical analyses were performed with SPSS (version 20.0; SPSS Inc., Chicago, IL, USA), and a *p* value <0.05 was considered significant.

### Ethical consideration

The SNUCM institutional review board approved the study protocol.

## Results

### Error disclosure performance of medical students and medical interns

Participant performance compared between medical students and medical interns showed no significant differences (Table 1). The average performance scores for medical interns and medical students were 3.067 and 2.950, respectively (*p* = 0.840). In particular, no participant explained the steps to take for prevention of future errors.

**Table 1:** Error disclosure performance rating scale score

	Response	Medical interns	Medical students	*p* value*
Number of participants with actual SP encounter		6	10	
Explanation of medical facts regarding error, n (%)	1	0	0	1.000
	2	0	0	
	3	4 (66.7)	6 (60.0)	
	4	2 (33.3)	4 (40.0)	
	5	0	0	
Honesty and truthfulness, n (%)	1	0	0	1.000
	2	0	0	
	3	3 (50.0)	6 (60.0)	
	4	3 (50.0)	4 (40.0)	
	5	0	0	
Empathy, n (%)	1	0	0	1.000
	2	0	0	
	3	3 (50.0)	4 (40.0)	
	4	4 (66.7)	6 (60.0)	
	5	0	0	
Prevention of future errors, n (%)	1	6 (100.0)	10 (100.0)	NA
	2	0	0	
	3	0	0	
	4	0	0	
	5	0	0	
General communication skills, n (%)	1	0	0	0.608
	2	0	0	
	3	2 (33.3)	6 (60.0)	
	4	4 (66.7)	4 (40.0)	
	5	0	0	

*P-values are calculated using Fisher’s exact test

### Response to medical errors, perceived responsibility, and their prevention

All six interns and all 79 medical student answered the questionnaire. Regarding the response to medical errors, most participants (100%, 78.8%, and 78.8% for cases 1, 2, and 3, respectively, *p* < 0.0001) answered that they would fully explain the situation and apologize about the error to patients and families. Participants’ feelings of duty about disclosing error decreased with decreasing severity of error outcome; scores for cases 1, 2, and 3 were 5.753, 4.827, and 4.463, respectively (*p* < 0.0001) (Table 2). Participants felt that they should disclose medical error and apologize for these errors when the outcome caused by the error is fatal. However, participants became more reserved when medical errors caused minor or no harm. There was no significant perceptional difference between medical students and medical interns, however. To prevent medical errors, an average of 88% of participants answered that changing the system is required in every case (90, 87, and 86% for cases 1, 2, and 3 respectively, *p* = 0.782).

**Table 2:** Participant’s Response to medical errors

	Response*	Case 1	Case 2	Case 3	*p* value**
Apologizing medical error to patient and family, n (%)	1	0	0	2 (3.5)	
	2	0	6 (7.5)	3 (3.7)	<0.0001
	3	0	7 (8.7)	8 (10.0)	
	4	2 (3.4)	12 (15.0)	22 (27.5)	
	5	17 (20.0)	27 (33.7)	33 (41.2)	
	6	66 (77.6)	28 (35.0)	12 (15.0)	

* 1 = strongly disagree; 2 = disagree; 3 = disagree a little; 4 = agree a little; 5 = agree; and 6 = strongly agree

** *P*-values are calculated using Fisher’s exact test

### Participant satisfaction with the education program

Most participants (79.7%) were satisfied with the education program, and medical interns were more satisfied than medical students (*p* = 0.042) (see Additional file 4). As for change after the education program, 65% of participants answered that they had become more confident in coping with medical errors through the simulated experience. They also thought that they should be prepared for and concerned about how to manage the situation. Comments about the education program were as follows:

- It was real! I could experience the situation, not just learn the situation.
- I became confident about dealing with difficult situations such as medical error disclosure.
- I came to realize the importance of communication with patients.
- I should deliberate about how to apologize for errors.
- I came to know that just apologizing doesn’t work.
- Legal advice should be added.

## Discussion

In this study, participants performed fairly well on several areas of error disclosure: explanation of medical facts regarding error, honesty and truthfulness etc. But their performances were unsatisfactory in some areas, particularly explaining future error prevention. Several reasons could explain participants’ unskilled performance in error disclosure. National guidelines for error disclosure have not yet been established in Korea, and lack of training or an education program may also have affected participant performance.

As there had been no regular curriculum with the subject of error disclosure in undergraduate medical education in SNUCM and early phase of medical internship program in SNH & CUH, our program was the first educational program for medical error disclosure using simulation to the participants who were all senior medical students or medical interns. Previous research supports the effectiveness of SPs for teaching error disclosure skills [6, 16, 19–21], and simulated encounter with SPs has been used to improve physician error-disclosing skills and confidence [19, 20]. Our program also used these simulations, and perceived participant confidence in understanding and performing full disclosure was improved.

In our study, performance between medical students and medical interns did not differ significantly. Because medical interns in our study were early in their medical internship (just before or after starting their internship) and had no experience or education program for disclosing medical error, the groups did not differ. Moreover, no participant who directly encountered SP explained the steps to take for prevention of future errors. We had a didactic session after rather than before the SP encounter, so participants had no idea about giving information for preventing future errors to patients and families. Although patients want to know that hospitals and physicians have learned from an event so that the error is not repeated, physicians rarely recognize the need of explaining and disclosing to patients about any efforts for preventing errors [15].

Disclosing medical errors to patients have ethical rationales such as informed consent, truth-telling, justice and fairness [7, 22–25]. Despite ethical rationales, a disclosure gap persists. A variety of studies have documented error disclosure rates of approximately 30% [8–12]. Various factors including fear of malpractice suit and dishonour of admitting an error to a patient are making physicians still hesitate to disclose errors to patients [26, 27]. Among them, fear of malpractice suit is a significant barrier for error disclosure. Although evidence supports that patients are more likely to sue physicians when there is no truthful communication, many physicians are afraid that disclosing medical errors to patients will precipitate lawsuits [28, 29]. Some hospital administrators and risk managers still say that physicians should not apologize to patients because an apology is regarded as an admission of fault [30]. Lack of formal training of disclosing medical error is one of the barrier for disclosure. If not trained properly, physicians don’t feel comfortable in conducting those conversations [18, 28].

Ethical complexities in error disclosure also account for disclosure gap. Often, there are uncertain situations whether unexpected outcome was caused by medical error. Furthermore, physicians tend to hesitate to disclose errors to patients when errors caused minor or no harm although they think fatal errors should be disclosed to patients. In addition, there has been little consensus about whether or not to disclose errors to patients when harmful errors involve patients who couldn’t live longer regardless of errors [25, 31]. In such situations, physicians think that disclosure can give no benefit to patients. In training program of disclosing medical error, learners should be trained to balance the ethical complexities related in error disclosure.

Our study also supports that participants felt little duty when the error caused minor or no harm whereas they felt that a fatal error should be disclosed. In addition to the error factors such as degree of harm caused by the error or patient awareness of error, institutional culture factor including supportive infrastructure and supposed tolerance for error, provider or patient factor influence the decision to disclose a medical error [32].

There are several limitations in our study. First, we had a small number of participants, so it is hard to generalize our results to other institutions. Second, for the medical students, we could not give all students the chance to have the SP encounter, and only a small number were able to do so. The other students observed and discussed the case. As there had been no error disclosure curriculum, students hesitated to participate in direct SP encounter. Thus, medical students who just observed the SP encounter might have benefited comparatively little from the curriculum. Third, we used one scenario in the simulation, so we cannot generalize this study result to other situations. Fourth, as we didn’t survey participants’ attitudes on medical error prior to the education program, it is hard to measure the change of the participants’ attitudes following the education program. Next time, we would try to make a better designed education program for medical error disclosure with pre and post survey based on current research.

## Conclusions

In conclusion, our error disclosure education program using simulation helped participants become confident about disclosing errors to patients and gave the participants important experience in thinking over medical errors and disclosing them to patients. Also we found that duty feelings of apology of medical students and medical interns vary according to the severity of the medical error outcome. Extending the program to more diverse scenarios and diverse groups of physicians (such as senior residents and faculties) is needed.

## Additional files

Additional file 1: Error scenario used during encounters between standardized patient and medical interns or medical students. (DOCX 15 kb)
Additional file 2: Items on the error disclosure rating scale for SP encounters. (DOCX 15 kb)
Additional file 3: Three clinical vignettes describing medical error. (DOCX 14 kb)
Additional file 4: Participant satisfaction with the education program. (DOCX 17 kb)
Additional file 5: Evaluation Form on the error disclosure performance during SP encounters. (DOCX 16 kb)
Additional file 6: Post-Education Program Survey. (DOCX 15 kb)
Additional file 7: error disclosure performance rating score. Description of data: Raw data of error disclosure performance data of 16 participants using rating scale. (XLSX 9 kb)
Additional file 8: Participant’s Response to medical errors. Description of data: Raw data of participant’s response to medical errors (3 clinical cases with different severity of error outcome), satisfaction and change after the education program. (XLSX 18 kb)

## Abbreviations

SP: Standardized patient
