# Supplementary material for: Improving disclosure of medical error through educational program as a first step toward patient safety
Source: BMC Med Educ. 2017 Mar 4;17:52. doi: 10.1186/s12909-017-0880-9 (PMC5336642; doi:10.1186/s12909-017-0880-9)
Supplement: Additional file 1: — Error scenario used during encounters between standardized patient and medical interns or medical students. (DOCX 15 kb) [file 12909_2017_880_MOESM1_ESM.docx]

**Additional file 1.** Error scenario used during encounters between standardized patient and medical interns or medical students

| You are the physician on an orthopaedic team. Mr. Lee is a 38-year-old patient who visited the orthopaedic outpatient clinic for wrist pain. Last night, he had slipped and developed right wrist pain. He had a medical history of drug allergy to NSAIDs and had already talked to someone on your team. After checking the X-ray of his wrist, you prescribed medication. He also wanted pain control because he said he couldn’t do his work due to pain.  After prescribing an NSAID injection, you learned that he had a medical history of drug allergy to NSAIDs.  Now he is complaining of chest tightness and pruritus. You are seeing him for the first time after the event to tell him what has happened. |
| --- |
