# Supplementary material for: Improving disclosure of medical error through educational program as a first step toward patient safety
Source: BMC Med Educ. 2017 Mar 4;17:52. doi: 10.1186/s12909-017-0880-9 (PMC5336642; doi:10.1186/s12909-017-0880-9)
Supplement: Additional file 2: — Items on the error disclosure rating scale for SP encounters. (DOCX 15 kb) [file 12909_2017_880_MOESM2_ESM.docx]

**Additional file 2. Items on the error disclosure rating scale for SP encounters**

Explanation of medical facts regarding error

- How did it happen?
  - Told me what the error was in my care
  - Explained to me why the error occurred
- What are the consequences?
  - Told me how the error impacted my health
  - Told me how the consequences of the error will be corrected
- Overall impression of explanation of medical facts regarding error

Honesty and truthfulness

- - Took responsibility for the error
  - Explained the error to me freely and directly, without my having to ask a litany of probing questions to get the details
  - Did not keep things from me that I should know
  - Never avoided my questions (not evasive)
- Overall impression of honesty and truthfulness

Empathy

- Apology—said he/she was sorry and apologized in a sincere manner with acknowledgement of feelings
  - Allowed me to express my emotions regarding this error
  - Told me that my emotional reaction was understandable
- Overall impression of empathy

Prevention of future errors

- - Told me that an effort will be made to prevent a similar error in the future
  - Told me what he/she would have done differently
  - Told me his/her plan for preventing similar errors in the future
- Overall impression of future errors

General communication skills

- - Degree of coherence in the interview
  - Verbal expression
  - Nonverbal expression
  - Responsive to my needs
  - Checked for my understanding of the information he/she provided
- Overall impression of general communication skills
