# Supplementary material for: Improving disclosure of medical error through educational program as a first step toward patient safety
Source: BMC Med Educ. 2017 Mar 4;17:52. doi: 10.1186/s12909-017-0880-9 (PMC5336642; doi:10.1186/s12909-017-0880-9)
Supplement: Additional file 4: — Participant satisfaction with the education program. (DOCX 17 kb) [file 12909_2017_880_MOESM4_ESM.docx]

**Additional file 4.** Participant satisfaction with the education program

|  | Response | Medical interns | Medical students | *p* value* |
| --- | --- | --- | --- | --- |
|  |  |  |  |  |
| Number of participants answered the Questionnaire |  | 6 | 73** |  |
| Satisfaction with the Education Program, n (%) | 1 | 0 | 2 (2.7) | 0.042 |
|  | 2 | 0 | 3 (4.1) |  |
|  | 3 | 0 | 11 (15.0) |  |
|  | 4 | 2 (33.3) | 36 (49.3) |  |
|  | 5 | 4 (66.7) | 21 (28.7) |  |

*P-values are calculated using Fisher’s exact test

** There was six censored case due to no answer.
