# Supplementary material for: Improving disclosure of medical error through educational program as a first step toward patient safety
Source: BMC Med Educ. 2017 Mar 4;17:52. doi: 10.1186/s12909-017-0880-9 (PMC5336642; doi:10.1186/s12909-017-0880-9)
Supplement: Additional file 5: — Evaluation Form on the error disclosure performance during SP encounters. (DOCX 16 kb) [file 12909_2017_880_MOESM5_ESM.docx]

| Component | Element | 1  poor | 2  Fair | 3  Good | 4  Very good | 5 Excellent |
| --- | --- | --- | --- | --- | --- | --- |
| Explanation of medical facts regarding error | - How did it happen?   - Told me what the error was in my care   - Explained to me why the error occurred - What are the consequences?   - Told me how the error impacted my health   - Told me how the consequences of the error will be corrected - Overall impression of explanation of medical facts regarding error |  |  |  |  |  |
| Honesty and truthfulness | - - Took responsibility for the error   - Explained the error to me freely and directly, without my having to ask a litany of probing questions to get the details   - Did not keep things from me that I should know   - Never avoided my questions (not evasive) - Overall impression of honesty and truthfulness |  |  |  |  |  |
| Empathy | - Apology—said he/she was sorry and apologized in a sincere manner with acknowledgement of feelings   - Allowed me to express my emotions regarding this error   - Told me that my emotional reaction was understandable - Overall impression of empathy |  |  |  |  |  |
| Prevention of future errors | - - Told me that an effort will be made to prevent a similar error in the future   - Told me what he/she would have done differently   - Told me his/her plan for preventing similar errors in the future - Overall impression of future errors |  |  |  |  |  |
| General communication skills | - - Degree of coherence in the interview   - Verbal expression   - Nonverbal expression   - Responsive to my needs   - Checked for my understanding of the information he/she provided - Overall impression of general communication skills |  |  |  |  |  |

**Additional file 5.** Evaluation Form on the error disclosure performance during SP encounters
