# Supplementary material for: Improving disclosure of medical error through educational program as a first step toward patient safety
Source: BMC Med Educ. 2017 Mar 4;17:52. doi: 10.1186/s12909-017-0880-9 (PMC5336642; doi:10.1186/s12909-017-0880-9)
Supplement: Additional file 6: — Post-Education Program Survey. (DOCX 15 kb) [file 12909_2017_880_MOESM6_ESM.docx]

**Additional file 6.** Post-Education Program Survey

<1> Medical Error Case

Case 1

You are the physician on a paediatric oncology team. JJ Lee is a 9-year-old boy who was diagnosed with ALL and admitted to the hospital for the last chemotherapy. He had completed multiple cycles of chemotherapy, and intrathecal methotrexate and IV vincristine treatment are scheduled. You injected vincristine into the intrathecal space. What would you do?

1) How would you manage this situation?

2) Would you apologize to the patient and family?

| 1  strongly disagree | 2  disagree | 3  disagree a little | 4  agree a little | 5  agree | 6  strongly agree |
| --- | --- | --- | --- | --- | --- |

3) Please provide a way to prevent this situation.

Case 2

You are the internist. SJ Park is a 62-year-old woman who had visited your clinic 3 days ago for fever and cough. After examining her, you thought that she had pneumonia and prescribed levofloxacin and azithromycin. She came again with no symptom improvement following taking the medications. Reviewing your previous order, you have found that you had ordered ‘azathioprine’ instead of ‘azithromycin’. What would you do?

1) How would you manage this situation?

2) Would you apologize to the patient and family?

| 1  strongly disagree | 2  disagree | 3  disagree a little | 4  agree a little | 5  agree | 6  strongly agree |
| --- | --- | --- | --- | --- | --- |

3) Please provide a way to prevent this situation.

Case 3

You are the physician on a paediatric emergency team. YG Lee is a 5-month-old baby who came to the emergency department with fever and vomiting. You ordered IV fluid for her due to fever and dehydration. During preparation for intravenous catheterization, she fell down when you were not holding her. Her parents are outside the room and are not aware of this situation. What would you do?

1) How would you manage this situation?

2) Would you apologize to the patient and family?

| 1  strongly disagree | 2  disagree | 3  disagree a little | 4  agree a little | 5  agree | 6  strongly agree |
| --- | --- | --- | --- | --- | --- |

3) Please provide a way to prevent this situation.

<2> Did you Satisfied with the Program?

| 1  very unsatisfied | 2  unsatisfied | 3  neutral | 4  satisfied | 5  very satisfied |
| --- | --- | --- | --- | --- |

<3> Please describe the change of your attitude or confidence regarding error disclosure after Education Program.
